# Effectiveness of Stress Shielding Prevention Using a Low Young’s Modulus Ti-33.6Nb-4Sn Stem: A 7-Year Follow-Up Study

**DOI:** 10.3390/medsci13020051

**Published:** 2025-05-01

**Authors:** Kazuyoshi Baba, Yu Mori, Hidetatsu Tanaka, Ryuichi Kanabuchi, Yasuaki Kuriyama, Hiroaki Kurishima, Kentaro Ito, Masayuki Kamimura, Daisuke Chiba, Toshimi Aizawa

**Affiliations:** Department of Orthopaedic Surgery, Tohoku University Graduate School of Medicine, Sendai 980-8574, Japan; kazuyoshi.baba.e3@tohoku.ac.jp (K.B.); hidetatsu.tanaka.c7@tohoku.ac.jp (H.T.); ryuichi.kanabuchi.b8@tohoku.ac.jp (R.K.); yasuaki.kuriyama.b5@tohoku.ac.jp (Y.K.); marronile@gmail.com (H.K.); itoken_319_rk@yahoo.co.jp (K.I.); masayuki.kamimura.b4@tohoku.ac.jp (M.K.); daisuke.chiba.a3@tohoku.ac.jp (D.C.);

**Keywords:** Ti-33.6Nb-4Sn (TNS), total hip arthroplasty, cementless stem, stress shielding

## Abstract

Background: Stress shielding (SS) after total hip arthroplasty (THA) leads to proximal femoral bone loss and increases the risk of complications such as implant loosening and periprosthetic fracture. While various low-stiffness stems have been developed to prevent SS, they often compromise mechanical stability. A novel femoral stem composed of Ti-33.6Nb-4Sn (TNS) alloy offers a gradually decreasing Young’s modulus from proximal to distal regions, potentially improving load distribution and reducing SS. This study aimed to evaluate the mid-term clinical and radiographic outcomes of the TNS stem, with a particular focus on its effectiveness in suppressing SS. Methods: A prospective clinical study was conducted involving 35 patients who underwent THA using the TNS stem, with a minimum follow-up of 7 years. Twenty-one patients with Ti6Al4V metaphyseal-filling stems served as controls. Clinical outcomes were assessed using Japanese Orthopaedic Association (JOA) scores, and radiographic SS was graded using Engh’s classification and analyzed in Gruen zones. Inter-examiner reliability and statistical comparisons between groups were performed using appropriate tests. Results: The TNS group showed significantly higher preoperative JOA scores than the control group, but no significant difference in final scores. Both groups demonstrated significant improvement postoperatively. Third-degree SS occurred in the TNS group, although the overall SS grade distribution was significantly lower than in the control group (*p* = 0.03). SS frequency was significantly reduced in Gruen Zones 2, 3, and 6 in the TNS group. Conclusions: The TNS stem demonstrated a significant reduction in SS progression compared to conventional titanium stems over a 7-year period, with comparable clinical outcomes. However, the occurrence of third-degree SS indicates that material optimization alone may be insufficient, highlighting the need for further design improvements.

## 1. Introduction

Total hip arthroplasty (THA) is an established surgical procedure to improve hip pain and daily activities. The global increase in the elderly population has led to an increase in the number of patients with osteoarthritis. In addition, younger patients are increasingly seeking to maintain active lifestyles and improve their quality of life [1,2]. The number of THA has been increasing and is predicted to continue rising in the future. Consequently, the number of revision THA is expected to increase as primary THA continues to increase [3,4]. However, revising the THA for poor bone quality remains technically demanding.

Stress shielding (SS) induces proximal femoral bone loss, which may contribute to implant loosening and an increased risk of periprosthetic fracture of the femur [5,6]. The mismatch of stiffness of the femur and stem altered the load transmission. Under non-physiological load distribution, bone remodeling occurred by Wolff’s law, leading to the development of SS [6]. The Young’s modulus of human bone ranges from 10 to 30 GPa, whereas that of Ti6Al4V, a commonly used stem material, is approximately 110 GPa [7,8,9].

Stems with reduced stiffness were developed to prevent SS. However, these stems were frequently associated with insufficient bone fixation, elevated bone–stem interface stress, and a higher risk of fatigue fractures, leading to early mechanical failure [10]. The development of stems required innovation to address two contradictory requirements: achieving sufficient stiffness to ensure load transfer and fixation while reducing stiffness to match that of bone to prevent SS [11].

Using numerical design methods, Kuiper and Huiskes addressed these requirements by proposing an optimized stem with a non-homogeneously distributed Young’s modulus. It was reported that the optimized stem could reduce interface stress by >50% compared with that of a stem homogeneously [12]. Moreover, stems with a gradual reduction in Young’s modulus from the proximal to distal regions have been hypothesized to achieve more physiological load transfer, minimizing stress concentrations at the bone–stem interface. Numerical simulations by Kuiper and Huiskes demonstrated that non-homogeneous elastic properties could reduce interface stress by more than 50% compared with homogeneously stiff stems [12]. Therefore, the TNS stem was designed to gradually reduce stiffness along its length, aiming to better replicate the natural strain distribution of the femur and mitigate stress shielding.

**Figure 1:**
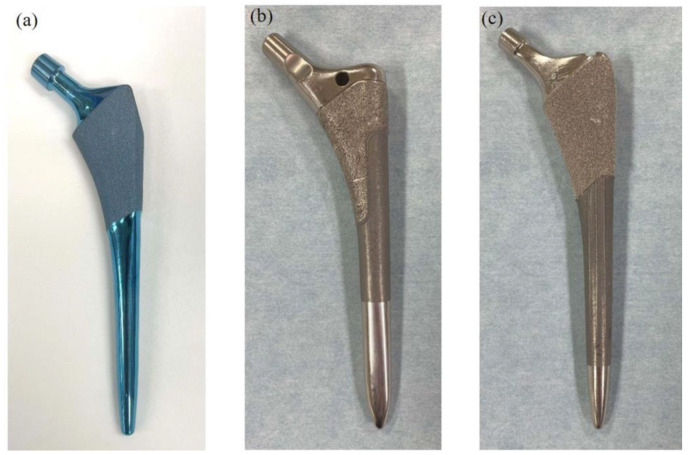
The morphology of stems implanted in this study. While the designs do not completely match, these stems are designed to fill the metaphysis. (**a**) TNS stem (Mizuho, Tokyo, Japan) (**b**) VerSys HA/TCP Fiber Metal Taper (Zimmer, Warsaw, IN, USA) (**c**) Synergy Select II (Smith & Nephew, Watford, England).

According to the theory, Hanada developed a femoral stem made of β-type Ti-33.6Nb-4Sn (TNS) alloy (Figure 1a). The Young’s modulus was changed gradually from proximal to distal [13]. Previous studies have reported that the TNS stem could prevent stem SS and offers comparable clinical outcomes to other fit-and-fill stems, contributing to improved hip pain and daily activities [14,15]. The TNS stem is a newly designed stem made of TNS alloy, though its mid- and long-term results remain uncertain. This study aims to evaluate the effectiveness of the TNS stem in reducing stress shielding and to assess its mid-term clinical and radiographic outcomes. 

## 2. Materials and Methods

### 2.1. Study Design

This is a prospective study conducted in a clinical trial from April 2016 to September 2017. This study was conducted in accordance with the Declaration of Helsinki and was approved by our institutional ethics board (No. 2021-1-1059). Informed consent was obtained from all patients.

### 2.2. Patients

Forty patients awaiting unilateral THA using a TNS stem were enrolled in the TNS group. Inclusion criteria were being over 20 years of age and having a diagnosis of osteoarthritis, avascular necrosis, or rheumatoid arthritis. Exclusion criteria were having had a previous operation on the affected side of the hip, arthroplasty, osteotomy, or tenotomy around the hip joint, bilateral hip disorder, rheumatoid arthritis of Charnley category C (multiple joint diseases or other diseases limiting mobility) [16], a history of deep venous thrombosis or pulmonary embolism, metal allergy, severe obesity (body mass index > 35.0 kg/m^2^), uncontrolled diabetes mellitus, or infection around the hip joint. Twenty-one patients who underwent THA in our department with a similarly designed Ti6Al4V stem and the same surgical approach from January 2007 to February 2011 were enrolled as a control group. The patients of the control group were diagnosed as having hip osteoarthritis or idiopathic osteonecrosis of the femoral head. Patients with unavailable data in medical records, poor-quality radiographs, or less than 5 years of follow-up were excluded.

### 2.3. Characteristics of the TNS Stem

TNS is a newly developed alloy with a low Young’s modulus of 40 GPa [13]. In addition to its low thermal conductivity, this alloy possesses unique properties, such as its Young’s modulus, and can be altered through heat treatment. The fabrication of the TNS stem through heat treatment has been thoroughly described in previous studies. In the present study, the TNS stem was fabricated in accordance with the method reported in those studies [11,13,17]. They were manufactured and provided by Mizuho Co., Tokyo, Japan. As briefly mentioned, localized heat treatment at the stem neck induced a temperature gradient, with higher temperatures proximally and lower temperatures distally along the stem. The heat treatment at 673 K for 5 h increased the Young’s modulus and tensile strength in the proximal region, gradually decreasing the Young’s modulus from the proximal to the distal end. The TNS alloy stem is a cementless tapered proximal fixation stem. The localized heat treatment induced a gradual change in the Young’s modulus along the stem, from approximately 70 GPa at the proximal part, to 50 GPa at the mid part, to 40 GPa at the distal tip. A schematic contour diagram illustrating this gradient change is provided in Figure S1 (Supplementary Materials). The TNS stem is shown in Figure 1a. The design of the TNS is categorized as a metaphyseal-filling stem [18]. The proximal one-third of the stem was treated with sandblasting, while the distal two-thirds were treated with polishing (Figure 1a).

### 2.4. Surgery and Rehabilitation

All patients of the TNS and control groups underwent THA with a posterolateral approach [19]. In the TNS group, a combination of the ARC HA cup and TNS stem was implanted. In the control group, patients received either a Trilogy cup with VerSys HA/TCP Fiber Metal Taper (Zimmer, USA) or a Reflection cup with a Synergy Select II (Smith & Nephew, England). The VerSys HA/TCP Fiber Metal Taper (Zimmer, USA) and Synergy Select II (Smith & Nephew, England) used in the control group were both made of Ti6Al4V and had designs similar to the TNS stem, classified as a metaphyseal-filling design (Figure 1b,c) [18]. All patients in both groups were allowed full weight-bearing from the first day after surgery.

### 2.5. Radiographic Evaluation

**Figure 2:**
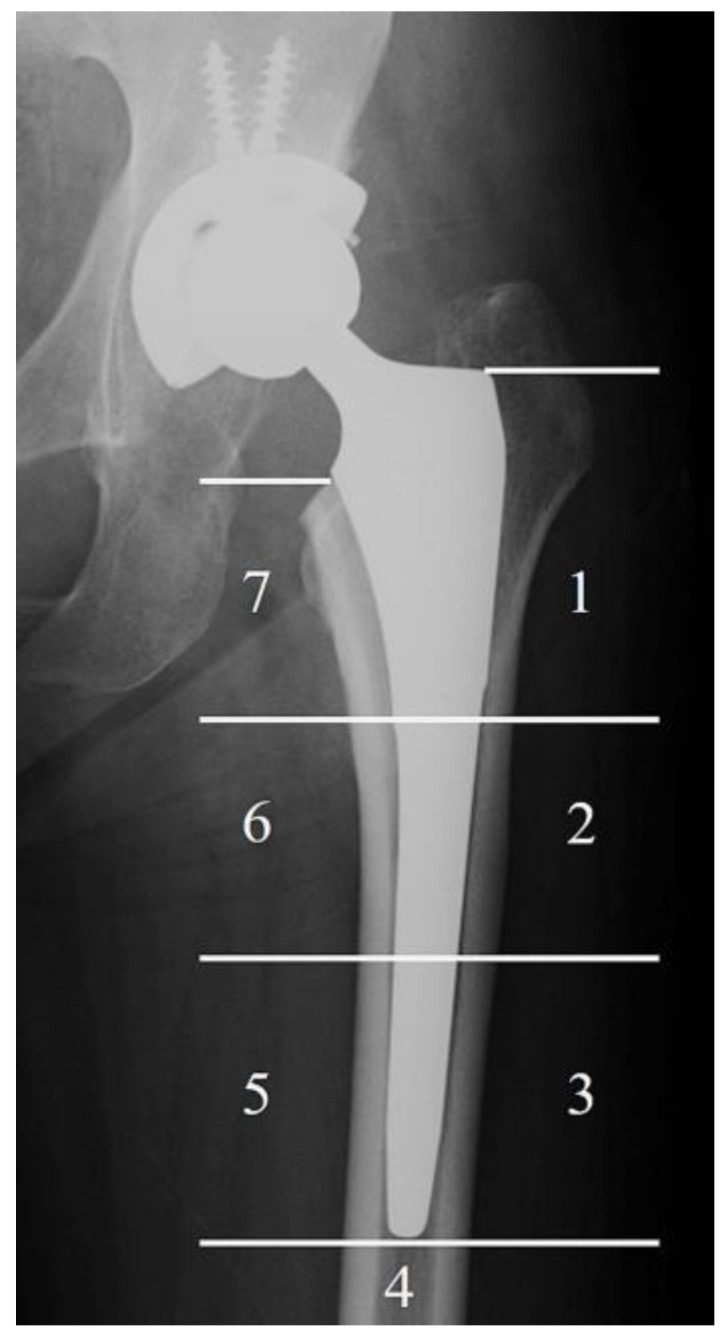
The Gruen zone. The bone resorption according to stress shielding was recorded by Gruen zone. The numbers show the area of the Gruen zone.

Anteroposterior radiographs of both hips and lateral radiographs of the affected hip were obtained immediately after surgery and at the final follow-up, which was more than five years post operation, in the TNS group. In the control group, radiographs were taken immediately after surgery and at a time corresponding to the postoperative observation period of the TNS group for comparison. SS was evaluated according to a previously reported method. [14]. The incidence of SS was assessed with radiographs at the final follow-up in both groups using Engh’s classification [20]. The frequency of SS at the final follow-up was also evaluated based on the Gruen zone (Figure 2) [14,21]. Two authors (K.B. and R.K.) who did not implant the stems performed radiologic assessments of SS independently under blind conditions.

Stress shielding was graded according to Engh’s classification [20], based on the extent of proximal femoral bone resorption:

Grade 0: No evidence of bone resorption.

Grade 1: Mild rounding of the proximal femoral neck without cortical bone loss.

Grade 2: Endosteal cortical resorption confined to the calcar region.

Grade 3: Progressive cortical bone resorption extending below the lesser trochanter.

Grade 4: Extensive cortical bone resorption along the stem.

### 2.6. Clinical Assessments

The Japanese Orthopaedic Association (JOA) scores were used to assess the clinical outcomes. The TNS group evaluated JOA scores preoperatively and at the final follow-up. In the control group, JOA scores were assessed preoperatively and at a follow-up time point matched to the postoperative observation period of the TNS group for comparison. The JOA hip scoring system is a 100-point scale that comprises subcategories of pain (40 points), range of motion (20 points), ability to walk (20 points), and activities of daily living (20 points) [22]. The occurrence of periprosthetic joint infection, periprosthetic fracture, dislocation, thigh pain, and the number of revision surgeries for any cause in the TNS group were evaluated based on their past medical record.

### 2.7. Statistical Analysis

The distribution of the continuous variable was assessed using the Shapiro–Wilk test. The continuous variables are expressed as mean ± standard deviation when normally distributed and as median (25–75 percentile) when non-normally distributed, respectively. Normally distributed continuous variables were analyzed using Student’s *t*-test, while non-normally distributed continuous variables were analyzed using the Wilcoxon signed-rank test. Gender and preoperative diagnosis were analyzed using Fisher‘s exact test. JOA scores were compared between TNS and control groups and between pre-surgery and final follow-up within each group. Depending on data distribution, Student’s *t*-test or Wilcoxon rank-sum test was used for group comparisons, and the paired *t*-test or Wilcoxon signed-rank test for within-group comparisons using JMP version 17 (SAS Institute Japan Ltd., Tokyo, Japan). Inter-examiner reliability of Engh’s classification grades was assessed using kappa coefficients, with statistical analyses conducted using R software (version 4.3.1; R Foundation for Statistical Computing, Vienna, Austria). Inter-examiner reproducibility was classified as follows: values < 0.50 indicated poor, 0.50–0.75 moderate, 0.75–0.90 good, and >0.90 excellent reliability [23]. Differences in SS incidence and its distribution across the Gruen zones were analyzed using Pearson’s Chi-square test. Statistical significance was set at *p* < 0.05. 

## 3. Results

### 3.1. Patient Demographics

Five patients in the TNS group were excluded due to a follow-up period of less than 5 years and insufficient clinical data. We enrolled thirty-two women and three men in the TNS group and eighteen women and three men in the control group. Table 1 shows the characteristics of the patients in this study. The mean age at surgery in the TNS and control group were 65.4 ± 7.7 and 62.6 ± 10.6 years, respectively. A combination of the VerSys HA/TCP Fiber Metal Taper and Trilogy cup was used in thirteen patients, and the Synergy Select II and Reflection cup was used in eight patients. The preoperative diagnosis was osteoarthritis in thirty-one patients, idiopathic osteonecrosis of the femoral head in four patients in the TNS group, hip osteoarthritis in seventeen patients, and idiopathic osteonecrosis of the femoral head in four patients in the control group. There were no significant differences in demographic data between the two groups (Table 1).

### 3.2. Radiographic Evaluation

One case in the TNS group was excluded from the assessment of SS due to revision THA performed for liner breakage. Osteolysis around the stem, caused by liner wear, may have influenced the evaluation of SS in this case. According to the Engh‘s classification, SS grades were distributed as follows: In the TNS group, 3 cases showed no SS, whereas first-, second-, third-, and fourth-degree SS were observed in nine, eleven, eleven, and zero cases, respectively. In the control group, first-, second-, third-, and fourth-degree SS were observed in five, six, five, and five cases, respectively. A weighted kappa value of 0.77 indicated substantial agreement. There was a significant difference in the distribution of SS grades between the TNS and control groups (*p* = 0.03) (Table 2).

Table 3 demonstrates the incidence of SS according to the Gruen zone. The overall inter-rater agreement between Examiner 1 and Examiner 2 across all Gruen zones (Zones 1 to 7) was moderate, with a Cohen’s kappa coefficient of 0.58. This indicates a fair level of consistency in the assessment of radiographic findings between the two examiners. SS was most frequently observed in Gruen Zone 7 in both the TNS and the control groups, while approximately half of the cases showed SS in Gruen Zone 1. Zones 2, 3, and 6 were the Gruen zones in which the TNS group demonstrated a significantly lower SS frequency than the control group (*p* = 0.02, 0.01, and 0.001 in zone 2, 3, and 6, respectively).

### 3.3. Clinical Assessment

**Figure 3:**
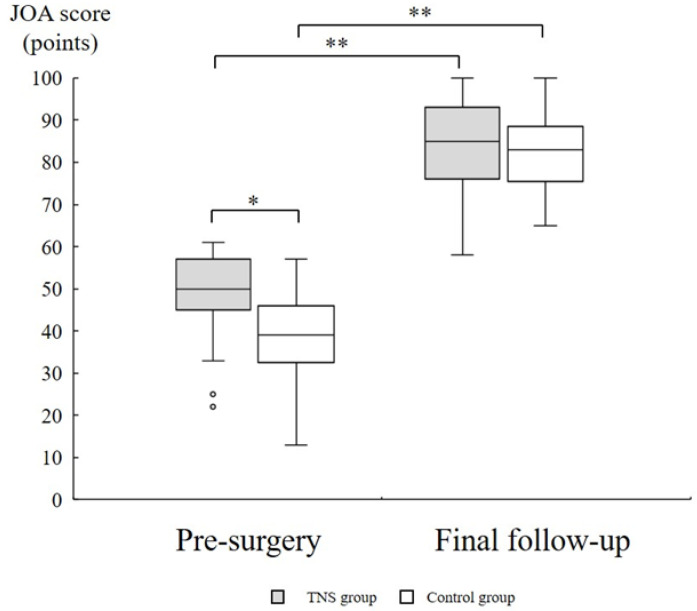
JOA score of pre-surgery and the final follow-up. Preoperatively, the TNS group showed significantly higher scores than the control group (Wilcoxon rank-sum test). At the final follow-up, no significant difference was observed (Student’s *t*-test). Within-group comparisons showed a significant improvement in JOA scores from preoperative to final follow-up (paired *t*-test or Wilcoxon signed-rank test, as variable distribution). *: *p* < 0.05; **: *p* < 0.01. JOA: The Japanese Orthopaedic Association.

Preoperative JOA scores were 50.0 (45.0–57.0) and 38.8 ± 11.2 in the TNS and control groups, respectively, demonstrating a statistically significant difference (*p* = 0.001). At the final follow-up, JOA scores were 84.6 ± 10.8 and 82.2 ± 9.8 in the TNS and control groups, respectively, with no significant difference observed (*p* = 0.41). Within-group comparisons revealed significant improvement after THA in both the TNS and control groups (*p* < 0.0001) (Figure 3). No cases of periprosthetic joint infection, periprosthetic fracture, or dislocation were reported in the TNS group. One TNS stem required revision THA due to liner breakage. Two patients in the TNS group reported thigh pain; however, the symptoms were mild.

### 3.4. Case Presentation

**Figure 4:**
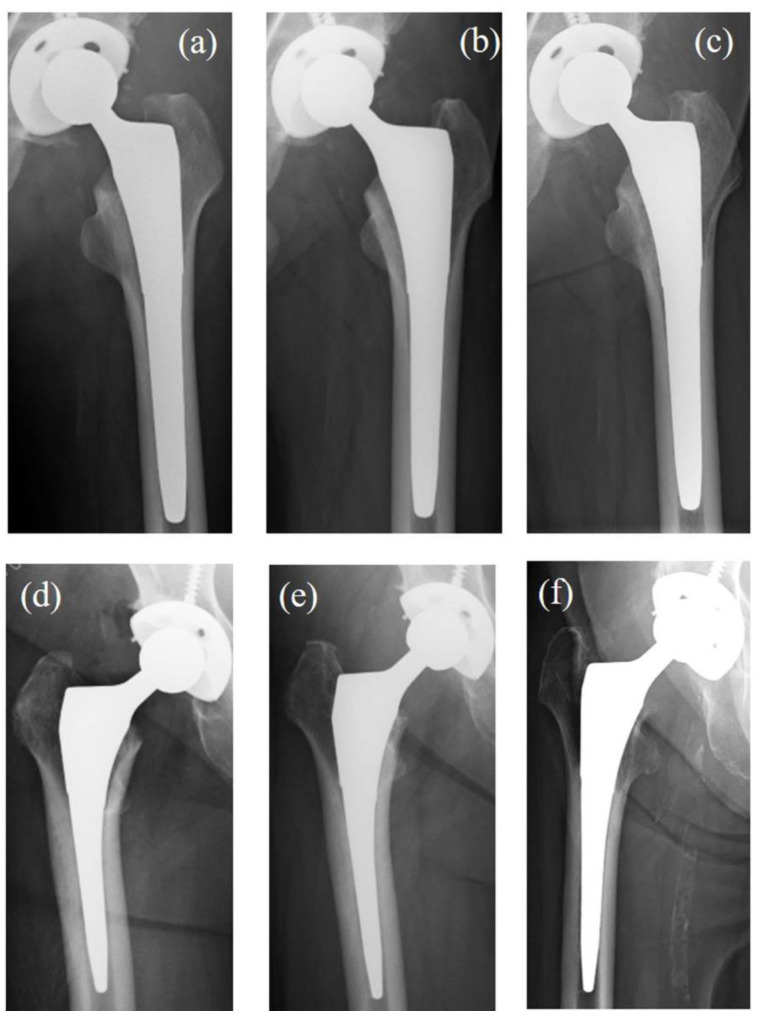
The X-rays of two cases of THA were performed using a TNS stem. Case 1 (**a**–**c**): A 58-year-old male diagnosed with idiopathic osteonecrosis of the left femoral head. No SS was observed at the final follow-up. (**a**) X-ray 3 weeks post operation. (**b**) X-ray 52 weeks post operation. (**c**) X-ray 94 months post operation. Case 2 (**d**–**f**): A 68-year-old female diagnosed with right hip osteoarthritis. Progressive SS was observed over time. (**d**) X-ray 3 weeks post operation. (**e**) X-ray 52 weeks post operation, showing rounding of the proximal femoral neck, consistent with first SS. (**f**) X-ray 85 months post operation, showing medial cortical bone resorption extending below the lesser trochanter, consistent with third SS. THA: total hip arthroplasty. SS: stress shielding.

Figure 4 presents two cases of THA performed using TNS stems: one without SS and one with progressive SS.

Case 1: A 58-year-old male diagnosed with idiopathic osteonecrosis of the left femoral head. No evidence of SS was observed at the final follow-up (Figure 4a–c).

Case 2: A 68-year-old female diagnosed with right hip osteoarthritis. At 52 months post operation, radiographic findings showed rounding of the proximal femoral neck, classified as first-degree SS. By the final follow-up, medial cortical bone resorption below the level of the lesser trochanter was noted, classified as third-degree SS (Figure 4d–f).

## 4. Discussion

This study investigated the radiographic and clinical performance of the TNS stem over a median follow-up of 7 years. The TNS stem showed a significant advantage in preventing SS compared to commercially available Ti6Al4V fit-and-fill stems, and its clinical outcomes were comparable. Notably, no implant failures directly attributable to the TNS stem occurred during the follow-up. In this series, no clinically significant stem subsidence was observed on follow-up radiographs. Minor initial subsidence, if any, did not appear to affect the evaluation of stress shielding or clinical outcomes. Previous studies have reported that the TNS stem effectively suppressed SS progression, with no observed cases of third- or fourth-degree SS, while achieving clinical results equivalent to Ti6Al4V stems of similar morphology [14,15]. However, these studies had a relatively short follow-up period of approximately three years. In the present study, although the progression of SS was significantly suppressed in the TNS group compared to the control group, third-degree SS was still observed. This delayed progression may be attributed to differences in strain distribution across the femur between the TNS and control stems. Regarding the progressive stress shielding observed in Case 2, possible contributing factors include suboptimal initial stem fit, patient-specific variations in proximal femoral bone quality, and altered postoperative loading patterns. These factors may have influenced the strain distribution around the femoral stem, thereby initiating remodeling processes leading to advanced stress shielding. However, the occurrence of third-degree SS even in the TNS group suggests that the gradient reduction in Young’s modulus within the TNS alloy alone is insufficient to fully prevent the progression of SS.

Previous studies utilizing bilateral THA models in dogs have demonstrated that femoral stems with lower stiffness, such as those made of titanium, result in significantly less SS compared to stiffer cobalt–chromium stems, even when evaluated at the same postoperative time point [24]. These findings suggest that increased stem stiffness may lead to earlier onset and more advanced progression of SS.

The difference in stiffness between the stem and the femur induces bone remodeling [13]. Mechanical loading on bone generates a strain stimulus [25,26]. Bone remodeling is initiated when the strain stimulus is reduced to levels lower than the physiological level to which the bone is normally adapted [27]. This stimulus depends on multiple factors, including magnitude, duration, loading rate, spatial distribution, and rest intervals [28]. In the TNS stem, the proximal stiffness mismatch with the femur was smaller than in the stems in the control group, keeping the strain stimulus near the adaptive level. Because it is defined by several factors, the stimulus may have occasionally been sufficient to initiate remodeling, and at other times not. This may explain the reduced SS and delayed progression of SS with the TNS stem.

In recent research, three-dimensional porous architectures, such as inner reticular or porous structures, have been adopted to reduce stem stiffness [5,29]. In contrast, the TNS alloy possesses a unique characteristic that gradually reduces Young’s modulus within a single material through heat treatment. However, this study suggested that the gradient change in Young’s modulus alone was insufficient to prevent the progression of SS. In addition to stiffness, other factors affecting SS include the stem cross-sectional geometry, stem length, and the degree of taper along the stem. Further research is needed to optimize the manufacturing method of the TNS stem. Although the stem offers a gradient change in Young’s modulus through heat treatment, this limits surface modification options, making it difficult to apply porous structures. Alternative methods, such as 3D printing, which do not require heat treatment, may be more suitable. Surface modification of the TNS alloy by anodic oxidation has also been reported to enhance osteoconductivity [30]. Improvement in osseointegration is considered important for the SS suppression. Potential design modifications to further suppress stress shielding include shortening the stem to facilitate load transfer closer to the metaphysis, optimizing the cross-sectional geometry to achieve more uniform strain distribution, and incorporating porous structures at the proximal region to promote bone ingrowth and reduce effective stiffness. Additionally, developing a modular design could allow better adaptation to individual femoral morphology, further improving load sharing.

Furthermore, the current design of the TNS stem is relatively long, making it less suitable for minimally invasive surgical approaches [31,32,33]. In addition, the bulky proximal portion lacks morphological compatibility with the femoral metaphysis, resulting in fixation predominantly at the proximal metaphyseal region and potentially contributing to the progression of stress shielding [34,35,36,37]. Accordingly, design modifications are warranted to enhance anatomical conformity and optimize load distribution.

There were several limitations in this study. First, the number of patients was small. The patient cohort included in the present analysis consisted of those originally enrolled in the prior clinical study, and no additional subjects were recruited thereafter. As a result, the sample size remained limited. Second, the morphology and surface treatment of the stems used in this study were not completely uniform. Third, objective assessment of bone remodeling was insufficient, as bone mineral density around the stems was not measured. We adopted the same qualitative approach for evaluating the immediate postoperative X-rays following THA in order to maintain methodological consistency with the previous study.

## 5. Conclusions

The TNS stem demonstrated a significant preventive effect against SS progression at 7 years post-THA compared to conventional titanium stems. In contrast, clinical scores were comparable between the two groups. No femoral stem-related failures were observed during the study period. Further research is warranted to develop improved stem designs or surface modifications to better address stress shielding. Although no femoral stem-related failures were observed during the 7-year follow-up, longer-term surveillance remains necessary to fully assess the durability of the TNS stem and the potential risk of late-onset complications such as fatigue fractures. Future studies with extended follow-up periods will be critical for validating these mid-term findings.

## Figures and Tables

**Table 1:** Demographics of patients.

	TNS Group	Control Group	*p* Value
Number of patients (hips)	35	21	
Gender, female: male, n (%)	32 (91): 3 (9)	18 (86): 3 (14)	*p* = 0.66
Mean age at surgery (years)	65.4 ± 7.7	62.6 ± 10.6	*p* = 0.26
Body mass index (kg/m^2^)	24.7 ± 4.1	24.4 ± 2.8	*p* = 0.78
Follow-up period (months)	87 (84–96)	91.8 ± 4.4	*p* = 0.27
Implant			
Stem, n	TNS stem, 35	Versys Taper, 13	
		Synergy Select II, 8	
Cup, n	ARC HA Cup, 35	Trilogy, 13	
		Reflection, 8	
Preoperative diagnosis, n (%)			
Osteoarthritis	31 (89)	17 (81)	*p* = 0.45
Osteonecrosis of the femoral head	4 (11)	4 (19)	

Data are expressed as mean ± standard deviation when normally distributed and as median (25–75 percentile) when non-normally distributed, respectively. Normally distributed continuous variables were analyzed using Student’s *t*-test, while non-normally distributed continuous variables were analyzed using the Wilcoxon signed-rank test. Gender and preoperative diagnosis were analyzed using Fisher‘s exact test. Statistical significance was *p* < 0.05.

**Table 2:** The distribution of SS in TNS and control group.

SS Grade	TNS Group	Control Group	*p* Value
0, n (%)	3 (9)	0 (0)	0.03 *
1, n (%)	9 (27)	5 (24)	
2, n (%)	11 (32)	6 (28)	
3, n (%)	11 (32)	5 (24)	
4, n (%)	0 (0)	5 (24)	

The analysis used Chi-square test. Statistical significance was *p* < 0.05. *: *p* < 0.05. SS: stress shielding.

**Table 3:** The incidence of SS according to Gruen zone.

Gruen Zone	TNS Group	Control Group	*p* Value
Zone 1, n (%)	15 (44)	10 (48)	1
Zone 2, n (%)	5 (15)	9 (43)	0.02 *
Zone 3, n (%)	0 (0)	4 (19)	0.01 *
Zone 4, n (%)	0 (0)	0 (0)	1
Zone 5, n (%)	0 (0)	1 (5)	0.38
Zone 6, n (%)	3 (9)	11 (52)	0.001 **
Zone 7, n (%)	27 (79)	17 (81)	1

The analysis used Fisher’s exact test or Chi-square test. Statistical significance was *p* < 0.05. *: *p* < 0.05; **: *p* < 0.01. SS: stress shielding.

## Data Availability

The data that support the findings of this study are available upon request from the corresponding author.

## Supplementary Materials

The following supporting information can be downloaded from the https://www.mdpi.com/article/10.3390/medsci13020051/s1. Figure S1: A schematic diagram illustrating the gradient of Young’s modulus. Reprinted with permission from [38].

## Abbreviations

The following abbreviations are used in this manuscript:

THA	total hip arthroplasty
SS	Stress shielding
TNS	β-type Ti-33.6Nb-4Sn
JOA	Japanese Orthopaedic Association
