# Supplementary figures and images for: Effectiveness of Stress Shielding Prevention Using a Low Young’s Modulus Ti-33.6Nb-4Sn Stem: A 7-Year Follow-Up Study
Source: Med Sci (Basel). 2025 May 1;13(2):51. doi: 10.3390/medsci13020051 (PMC12101376; doi:10.3390/medsci13020051)

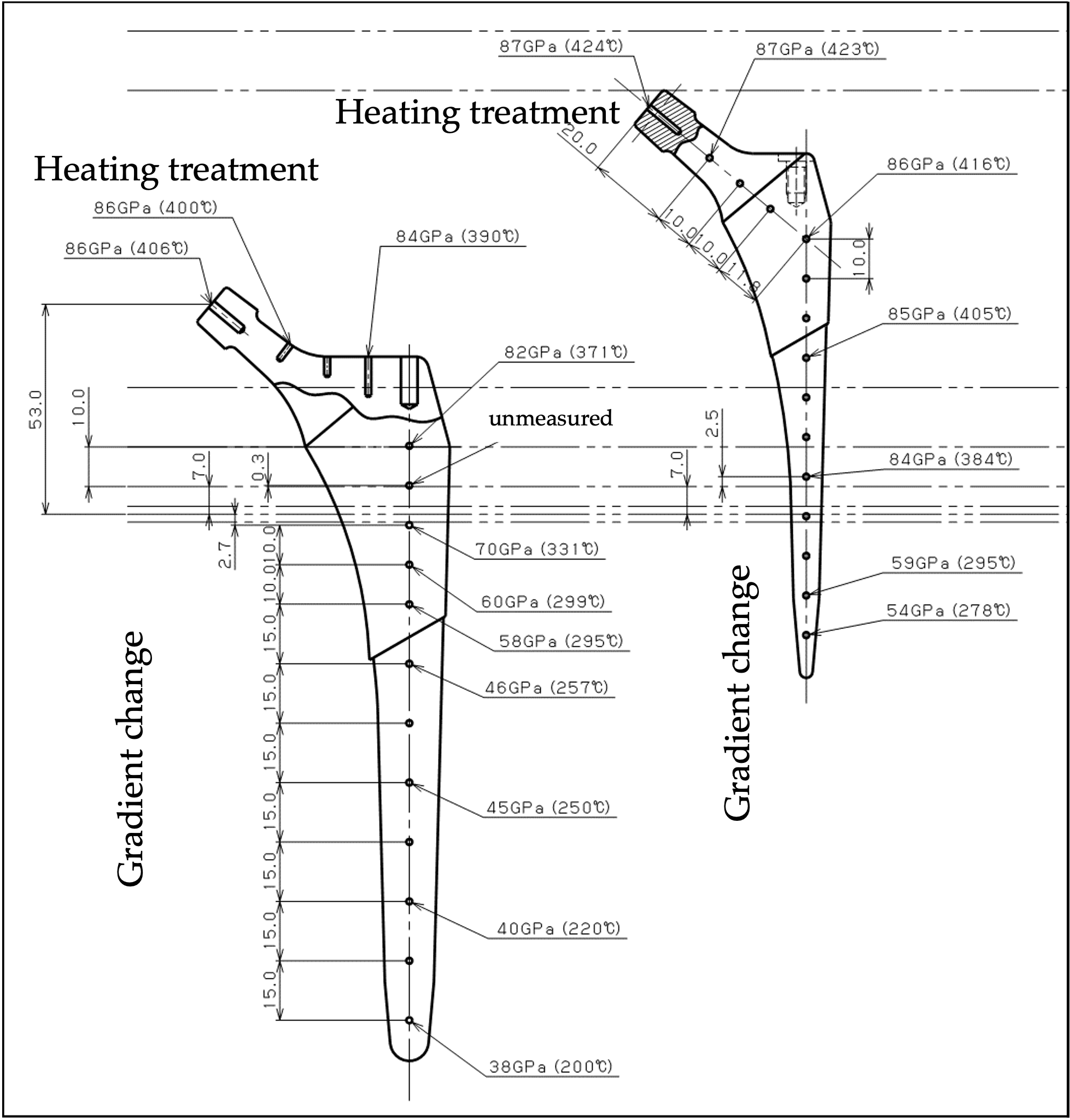

Supplement: Supplementary file 1 [file medsci-13-00051-s001.zip › medsci-3579647-supplementary.tiff]
